# Exploring Moral Injury Among Staff Involved in the Use of Seclusion Within Intellectual Disability Services

**DOI:** 10.1192/bjo.2026.11595

**Published:** 2026-06-30

**Authors:** Azeez Razaq-Oyetola, Larteque Lawson

**Affiliations:** Bradford District Care NHS Foundation Trust, Bradford, United Kingdom

## Abstract

**Aims:**

The use of seclusion in intellectual disability services presents significant ethical and emotional challenges for staff. While the impact of restrictive practices on patients has been widely explored, there is limited literature examining the potential for moral injury among staff involved in delivering seclusion. This service evaluation aimed to explore the nature of moral injury experienced by staff involved in seclusion within an intellectual disability assessment and treatment unit (ATU), alongside perceived contributing factors, available support, and coping strategies.

**Methods:**

An exploratory mixed-methods service evaluation was conducted in an intellectual disability assessment and treatment unit. Anonymous staff surveys were used to collect both quantitative and qualitative data. Quantitative data were gathered using a modified moral injury rating scale to explore levels of moral distress and wellbeing. Qualitative data were obtained through free text responses exploring staff experiences of seclusion, including ethical dilemmas and emotional responses. Quantitative data were analyzed descriptively, and qualitative data were analyzed using thematic analysis.

**Results:**

Findings suggest that staff involved in the use of seclusion may experience features consistent with moral injury. Key themes included the emotional impact of enforcing restrictive practices, perceived lack of autonomy within organisational systems, repeated or prolonged use of seclusion, and limited opportunities for structured debriefing and reflective practice. Staff described tensions between professional values and organisational demands, alongside variable access to psychological and supervisory support.

**Conclusion:**

This service evaluation highlights moral injury as a potential occupational risk for staff involved in seclusion within intellectual disability services. Addressing moral injury through reflective spaces, supervision and organisational support may be important in promoting staff wellbeing, reducing burnout, and maintaining standards of patient care. Further work is warranted to better understand moral injury in restrictive practices and to evaluate interventions aimed at mitigating its impact.

